# Spermatogenesis recovery treatment in less than four months from zero to almost 16 million sperms per milliliter after several cycles of steroids in 44‐year‐old healthy man

**DOI:** 10.1002/ccr3.8159

**Published:** 2023-11-06

**Authors:** Rade Injac

**Affiliations:** ^1^ Faculty of Pharmacy, The Chair of Pharmaceutical Biology University of Ljubljana Ljubljana Slovenia

**Keywords:** male fertility, spermatogenesis, steroids

## Abstract

The most common external factors that men can have influence on, and improve its own spermatogenesis, are related to lifestyle, habits, stress level, sport activities, nutrition quality, and medications. Steroids became one of the key issues in young men during reproductive stage of life. More and more teenagers who are not even developed yet naturally start using supplements very early to build up body and strengths of muscles in the shortest possible time. In this report is presented the case of 44‐year‐old (November 2022), healthy, and sporty man, who is 1–2 times per year on steroid cycles since he turned 40th. He had intention to become a father; however after 6 months of the last steroid cycle, sperm count was zero. This case will show what was needed and successfully done within less than 4 months after last steroids administration and the moment when sperm count reached almost 16 million sperms per milliliter. However, it has to be clear that this is unique case and additional tests would be needed on bigger population to confirm fast post steroid sperm count recovery in healthy subjects, using approach from this manuscript.

## Graphical abstract

Spermatogenesis recovery process in 44‐year‐old male after several cycles of steroids and fitness supplements. Timeframe of activities including basic body and healthy data from the case.

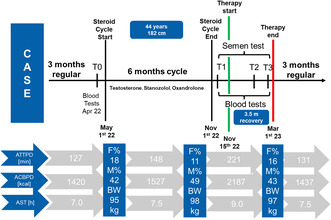

## 1 INTRODUCTION

Men fertility became one of the biggest health problems in the modern world. Different scientific groups would claim many factors which might be the key to create issue, which is for young couples planning the family, big frustration.^1^, ^2^ It is well known that throughout the past century stress,^3^ alcohol, drugs abuse, smoking,^4^ irregular sleeping habits,^5^ non‐balanced diet,^6^, ^7^ and many other external factors can have influence on spermatogenesis. Genetic factors^8^, ^9^, ^10^ and the one which are connected to some diseases^11^ having fertility consequently, we cannot influence easily or at all. Other factors which are external we can have under personal control in most of the cases. Hormones are big communal and health hazard which can create issues on the long term. It is very popular among women in reproductive era in modern part of the world to take oral contraceptives.^12^ These medications are based on female sexual hormones and after its metabolization, normally via urine it is finally released in communal water. In the regular and natural water cycle, these metabolites will get into the drinking water. Over the time with daily uptake of tap water, male population is exposed to female sexual hormones, which have negative influence on male development in puberty and spermatogenesis in reproductive ages. This external factor with oral contraceptives is very difficult to be controlled directly by male population itself.^12^ On the contrary, steroids,^13^, ^14^, ^15^ and food supplements to support bodybuilding on the fast track, are extremely popular nowadays among youngsters (to get shape and be large as soon as possible) and middle‐aged men (to keep youngsters body shape, strengths, and libido). In any case, use of anabolic steroids has direct and negative influence on spermatogenesis which was proven long time ago. Most of the scientific data are supporting reversibility of spermatogenesis in healthy men after steroid cycles.^13^, ^14^, ^15^ However, depends on the age, time that steroids have been used, amount and type of steroids, damage that has be done, and overall condition of the person, spermatogenesis can be back to normal level (above 15 million sperms per milliliter) within 12–24 months after last uptake of steroids^13^, ^14^, ^15^ or to limited level (below 15 million sperms per milliliter) without further improvements. In the first case fertilization on the natural way would be possible, while in the second case IVF is recommended if sperm count is at least 1 million per milliliter or above.

Many different approaches have been used in the past, including medications and food supplements (mostly antioxidants) to improve sperm count in healthy males.^13^, ^14^, ^15^, ^16^, ^17^, ^18^, ^19^, ^20^, ^21^, ^22^

In this paper, we report the case of the healthy, sporty, intellectual, educated (pharmacist), and very active man in his early 40th. His example was unique due to very fast and unusually successful level of spermatogenesis recovery after several steroids' cycles (4 months after last steroid use and 3.5 months after therapy and habit changes).

All literature data support tests done only using one parameter in clinical studies such as medications, antioxidants, different diet, habits changes, and sports; however, this is the first case where all of it was applied in one subject at the same time.

Patient gave consent for using his clinical data in research.

## 2 CASE

**FIGURE 1:**
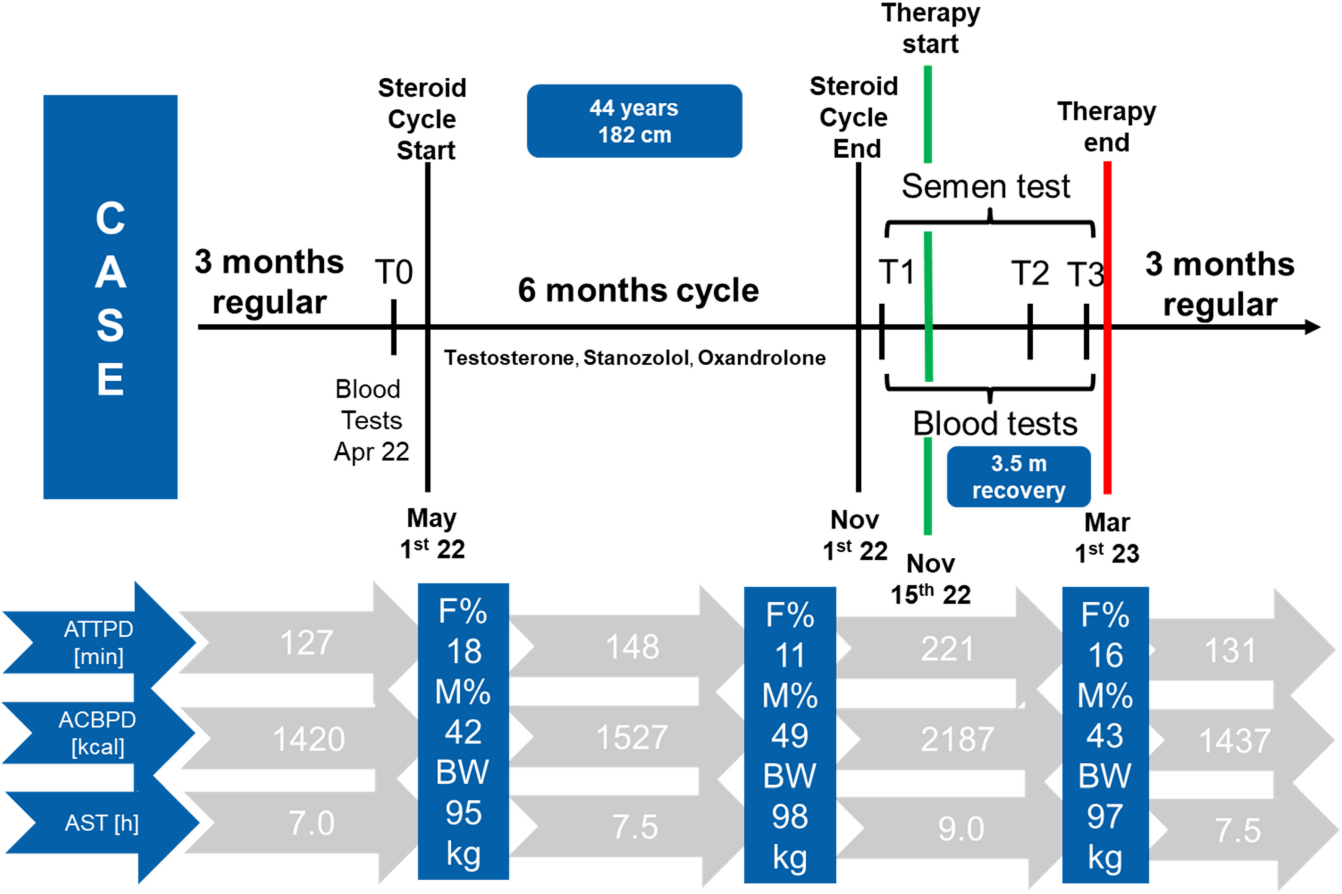
Case overview with testing, physical conditions, and activities prior and after last steroid cycle, including post treatment and habits changes. ATTPD—average training time per day; ACBPD—average calories burned per day; AST—average sleeping time; F%—body fat percentage; M%—muscular percentage of the body; BW—body weight.

A 44‐year‐old (November 2022) man (no children) presented to Fertility Center (Miami, FL, USA) with intention of having family using IVF approach, since natural way did not work out in past few years. His past medical history and physical examinations were all normal (Table 1). At the age of 40, he had on request of his GP within one‐year complete checkup including: dermatology, ophthalmology, dental care, pulmonology, gastrologic exams, cardiovascular tests, and urological investigations. All of them have been performed in Mount Sinai Medical Center (Miami Beach, FL, USA), and no issues have been found. In parallel the same year, on advice of endocrinologist after blood tests focusing hormones, patient got recommendation to start substitutional testosterone (depo form 150–250 mg per week) and human growth hormone therapy (1–3 UI per day). Following that recommendation at age of 40, patient started with steroid cycle and until today he did it in total 9 cycles (each ca. 4–6 months) before the time point T1 (Figure 1). The main reason for its start was to support better muscular endurance due to intensive trainings related to preparation for triathlon recreational competitions. Competitions were seasonal and patient used supplements only in preparation phase to increase muscular percentage and power.

**TABLE 1:** Medical history (key events).

Age	Month/Year	Medical status
0	November 1978	Birth—healthy male baby
0–15	1978–1993	All mandatory vaccinations for children (by Yugoslavian law)
1–12	1979–1990	Chronic bronchitis
7–30	1985–2008	Competitive swimmer
13	April 1991	Mumps virus—recovered without any consequences
17	April 1995	Tetanus vaccination (also in 2005 and 2015)
18	November 1996	Sperm count test—healthy (>15 million/mL)
20	January 1998	HPV infection—recovered within 1 year
22	October 2000	Seasonal flu vaccination (every year since 2000 until present time)
30	March 2008	Systemic candidiasis—recovered within 6 months
32	May 2010	Obesity (BMI 39; weight 130 kg; fat 37%); high cholesterol and blood pressure
32	October 2010	Hepatitis A and B vaccination (followed by second and third doses)
32	October 2010	Tick‐borne encephalitis vaccine (also November 2010, March 2012/2015/2020)
35	November 2013	Syphilis infection (successfully treated)
35–44	2013–2022	Healthy sporty conditions
39	October 2017	Left knee meniscus surgery
40	November 2018	Sperm count test—healthy (>15 million/mL)
40	November 2018	Steroid supplements (triathlon recreational competitions)—used till November 2022
40	December 2018	Gonorrhea infection (successfully treated)
41	2019	July and December—HPV vaccination (first and second doses)
42	March 2020	First Covid infection; second in December 2020; third in January 2022
42	October 2020	Syphilis infection (successfully treated)
43	2021	Covid vaccine—Sinopharm China—May 1; June 2; March 3, 2022
43	2021	Covid vaccine—Moderna Spain—September 1; October 2; June 3, 2022
44	June 2022	Monkeypox infection (successfully recovered)
44	November 2022	Sperm count test—azoospermia

During the lifetime, he did not have any diseases which might influence sperm production. (Table 1). At age of 18 (in Serbia) and 40 (in USA), he had spermatogenesis tests which have been both concluded as healthy and normal. Between age of 40 and 44, sperm count test was not performed until November 2022. After genetic, blood, and sperm tests in Fertility Center Miami, all 283 genetic diseases checked have been negative, and almost all blood tests (Table 2) have been within the normal range for adult man, except that spermatogenesis test showed zero sperms in semen sample (Table 3).

**TABLE 2:** Blood test results over 4 months period.

Parameter	End‐April 2022 T0	Mid‐November 2022 T1	End‐January 2023 T2	Beg‐March 2023 T3	Unit	Normal range
Leucocytes	4.99	5.12	5.20	4.92	g/l	3.5–10.0
Erythrocytes	5.54	5.43	4.79	5.98	t/l	4.5–6.3
Hemoglobin	169	174	163	165	g/l	140–180
Hematocrit	0.45	**0.54+**	0.48	0.51	l/l	0.38–0.52
MCV	92	**99+**	**100+**	94	fl	79–95
MCH	29.8	32.1	**34.1+**	33.0	pg	27.0–33.2
MCHC	340	325	340	335	g/l	320–360
Thrombocyte	157	191	**135‐**	151	g/l	150–450
Neutrophiles total	65.9	57.6	66.6	67.2	%	40.0–74.0
Neutrophiles absolute total	3.01	2.95	3.47	3.05	g/l	1.300–6.700
Lymphocyte	23.2	32.1	24.2	22.9	%	19.0–48.0
Lymphocyte absolute	1.37	1.64	1.26	1.32	g/l	0.900–3.300
Monocyte	5.4	6.2	4.6	5.2	%	3.4–9.0
Monocyte absolute	0.25	0.32	0.24	0.22	g/l	0.120–0.620
Eosinophile	2.8	2.1	3.0	2.7	%	0.0–7.0
Eosinophile absolute	0.15	0.11	0.16	0.13	g/l	0–0.300
Basophile	0.2	0.2	0.2	0.2	%	0.0–1.5
Basophile absolute	0.01	0.01	0.01	0.01	g/l	0–0.090
Natrium	140	136	139	138	mmol/l	135–145
Kalium	4.2	4.7	3.9	4.3	mmol/l	3.6–4.8
Chloride	102	101	104	101	mmol/l	97–110
Calcium	2.34	2.20	2.21	2.22	mmol/l	2.10–2.65
Phosphate	1.11	0.83	1.00	1.03	mmol/l	0.80–1.50
Creatine	97	**125+**	**109+**	96	μmol/l	49–97
Urea	6.8	6.9	6.8	7.0	mmol/l	3.2–7.3
Uric acid	333	317	349	327	μmol/l	258–491
Bilirubin	12.8	13.9	10.9	11.2	μmol/l	<24
AST	25	**50+**	32	27	U/l	11–34
ALT	22	**60+**	33	29	U/l	9–59
GGT	28	18	25	20	U/l	12–68
Protein total	75	73	68	70	g/l	64–83
Albumin	43	38	38	39	g/l	35–52
C reactive protein	1.0	2.1	<0.3	1.1	mg/l	<10.0
Alkaline phosphatase	81	107	74	77	U/l	40–130
LDH	195	181	197	190	U/l	135–225
Pancreas amylase	24	22	18	23	U/l	13–53
CK	193	**341+**	**213+**	198	U/l	50–200
CK‐MB mass	1.9	2.9	2.0	1.9	μg/l	<5.0
Testosterone	489	**1355+**	351	527	ng/dl	300–1100
Growth hormone	0.6	0.5	0.7	0.7	ng/ml	0.4–10.0
HIV test	Negative	Negative	Negative	Negative	N/A	Negative

Bold with +: values above the normal range.

**TABLE 3:** Sperm count results over treatment period.

Parameter/And timing	Mid‐November 2022 T1		End‐January 2023 T2		Beg‐March 2023 T3		Normal value
	Pre‐Thaw	Post‐Thaw	Pre‐Thaw	Post‐Thaw	Pre‐Thaw	Post‐Thaw	
Volume	NA	NA	2.5	0.1	3.0	0.1	>1.5 mL
Viscosity	NA	NA	Slight	Slight	Slight	Slight	/
Agglutination	NA	NA	Slight	Slight	Slight	Slight	/
Concentration	NA	NA	3.7	4.0	15.5	20.0	>15 mill/ml
Total count	NA	NA	9.2	0.2	46.5	2	>39 million
Motility	NA	NA	18	15	58	50	>40%
Progression	NA	NA	13	12	48	43	>32%
Total motile sperm	NA	NA	1.7	0.1	27	1	>20 million
Round cell	NA	NA	<3	NA	<3	NA	<3 million
pH	NA	NA	9.0	NA	8.7	NA	≥7.2
IgA	NA	NA	0	NA	0	NA	≤50%
IgG	NA	NA	0	NA	0	NA	≤50%
Leucocytes	NA	NA	0	NA	0	NA	<1 mill/ml
Granulocytes	NA	NA	0	NA	0	NA	<1 mill/ml

Complete picture of the activities of the patient before, during, and after steroid cycle is shown in the Figure 1. Test time points are also lister as following: T0—before steroid cycle, T1—just after steroid cycle, T2—control point 2.5 months after the therapy (habit changes), and T3—end of the therapy. Spermatogenesis tests been performed at 3 time points (T1, T2, and T3). T1 and T3 have been done in Fertility Center Miami (FL, USA), while T2 has been done in Fertisuesse Olten (Switzerland, Europe).

Since October 2017 (Table 4), patient is taking once per day Prep pill (Mount Sinai Medical Center—Miami Beach, FL, USA; 2017–2019) as part of HIV prevention, and accordingly, regular blood tests (Table 2) are done every 3 months in University Clinic Basel, Department for Infectiology (Switzerland, Europe; 2020–2023).

**TABLE 4:** Anabolic steroid cycle and post cycle semen recovery treatment.

Period	Treatment	Medications
Since Oct 2017	HIV prevention	Truvada® daily (PreP) Tenofovir disoproxil 245 mg + Emtricitabine 200 mg tablet p.o.
From May 1, 2022, to November 1, 2022 (6 months)	Anabolic Steroids Cycle	Testosterone depo injection i.m. 250 mg every fifth day Stanozolol tablet 20 mg p.o. 1/day Oxandrolone tablet 15 mg p.o. 1/day Anastrozole tablet 1 mg p.o. 1/day One A Day Men's tablet p.o. 1/day Omega‐3 (EPA + DHA 360 mg) soft capsule p.o. 1/day Vitamin C 1000 mg tablet p.o. 1/day
From November 10, 2022, to March 10, 2023 (4 months)	Semen Recovery Treatment	Clomiphene citrate tablet 50 mg p.o. 1/day One A Day Men's tablet p.o. 1/day Omega‐3 (EPA + DHA 360 mg) soft capsule p.o. 2/day Vitamin C 1000 mg tablet p.o. 1/day Vitamin C 1000 mg retard tablet p.o. 1/day Calcium 600 mg + D3 20 μg tablet p.o. 1/day Se 100 μg + C 90 mg + E 41 mg + A 450 μg capsule p.o. 1/day Vitamin E 100 mg p.o. 1/day CoQ10 50 mg + E 10 mg + A 800 μg + Se 80 μg capsule p.o. 1/day Fertil Aid® ‐ vitamins, minerals, amino acids, herbals; capsule p.o. 1/day Count Boost® ‐ C 500 mg + B2 15 mg + B3 25 mg + B12 500 μg + herbals; capsule p.o. 1/day Motility Boost® ‐ B6 25 mg + B12 500 μg + Amino acids + CoQ10 + herbals; capsule p.o. 1/day

After discussion and recommendations from the doctor, patient created his own plan of activities, including supplemental therapy to improve sperm count, mobility, and quality, in faster than 1 year (as doctor estimated to be needed). Complete list of medications during steroid cycle and during semen recovery treatment period are listed in Table 4, as well as below with the information of producers:

1. Truvada® Tenofovir disoproxil 245 mg + Emtricitabine 200 mg tablet, Gilead (Foster City, USA).
2. Testosterone depo injection, Galenika AD (Belgrade, Serbia).
3. Winstrol® Stanozolol tablet 20 mg, Prime Labs (Draper, USA).
4. Anavar® Oxandrolone tablet 15 mg, Prime Labs (Draper, USA).
5. Arimidex® Anastrozole tablet 1 mg, Astra Zeneca (London, UK).
6. One A Day Men's tablet, Bayer (Whippany, USA).
7. Omega‐3 (EPA + DHA 360 mg) soft capsule, Nature's Bounty (Bohemia, USA).
8. Vitamin C 1000 mg tablet, CVS Health (Woonsocket, USA).
9. Clomiphene citrate tablet 50 mg, Anfarm Hellas (Athens, Greece).
10. Vitamin C 1000 mg retard tablet, Goodwill Pharma (Szeged, Hungary).
11. Calcium 600 mg + D3 20 μg tablet, Finest Nutrition (Chattanooga, USA).
12. Oligogal SE® Se 100 μg + C 90 mg + E 41 mg + A 450 μg capsule, Galenika AD (Belgrade, Serbia).
13. Sant‐E‐Gal® Vitamin E 100 mg, Galenika AD (Belgrade, Serbia).
14. Biostile Q10 + Se®, CoQ10 50 mg + E 10 mg + A 800 μg + Se 80 μg capsule, Biostile (Komen, Slovenia).
15. Fertil Aid®—Fairhaven Health (Middletown, USA).
16. Count Boost®—Fairhaven Health (Middletown, USA).
17. Motility Boost®—Fairhaven Health (Middletown, USA).

At the same time in the Figure 1 is possible to see how before, during, and after the cycle (therapy), several parameters have been changing, such as active training time, calories burn rate, sleeping time, body fat, muscular percentage, and body weight.

It was clear from the beginning that longer therapy (max 3–4 months) would not be possible since some of the antioxidants and vitamins could damage liver in long‐term high‐dose usage.

## 3 DISCUSSION

Patient was using steroids between age of 40 and 44 (2018–2022). He was also trying on the natural way to get baby during this period (sperm count tests have not been performed). However, he was aware that supplements could influence sperm count. Therefore, he went to professionals to check whether IVF would be an option since above 1 million per milliliter sperm count is enough for IVF. At the end during checkup and consultations at age of 44 (November 2022), his sperm count was zero. Accordingly, no IVF was possible. Since patient was very healthy without any issues, focus was on supplements and steroids use as temporary issue.

Mechanism of action related to testosterone in male body, and it is correlations with spermatogenesis, hypothalamus, and anterior pituitary, is very well known.^15^ Within a very short period of only few months of anabolic steroids use major side effects can occur.^13^, ^14^, ^15^, ^23^ The purpose of this case report is focus on spermatogenesis and blood tests parameters changes due to negative effect of testosterone, stanozolol, and oxandrolone. Almaiman et al.^23^ reported most significant blood test parameters changes during anabolic steroids use. Already after several weeks and months of use, parameters typical for kidney, heart, and liver will be higher than normal range. In this case, we can see in the Table 2 that same parameters have been above normal level which is typical after use of testosterone and/or oral dihydrotestosterone‐like effects steroids (stanozolol and oxandrolone). Creatine, AST, ALT, CK, and testosterone have been above normal range 2 weeks after last use of steroids. Already after 3.5 months of recovery with therapy and new habits, all of them came back to normal level.

Even though that in literature there are some recommendations related to therapy with prescribed medications in case of post cycle recovery period,^13^, ^14^, ^15^ patient in consultation with doctors choose to go only with one prescribed drug (Clomiphene Citrate) at the dose of 50 mg per day during the whole period of recovery. According to literature and practical experiences, normal production of healthy sperms after steroid's use is having recovery period of 12–14 months.^15^ To speed up this process, recommendation is to use clomiphene (25 or 50 mg daily) and human chorionic gonadotropin (daily dose 500–3000 UI) as combined therapy with minimum of 3 months treatment to optimal of 6–12 months.^13^, ^14^, ^15^ Since clomiphene was showing improvement in sperm quality in almost 60% of patient in previous studies,^16^ and combined treatment had around 67% of improvement in the period of 6 months, decision was to use only clomiphene.

In addition to this, patient made full program of habit changes as well and food supplement use (over the counter products) mostly antioxidants to jointly influence spermatogenesis. Prior to azoospermia (November 2022), patient used only 3 food supplements regularly as stated in Table 4. He is in the age of 44, which could be already critical for quality of sperm according to Jimbo et al.^10^ However, taking under consideration all medical exams done, as well as body shape and physical activities (Figure 1), patient had very healthy lifestyle and was in a good condition. In addition to medication, patient worked on addictive habits (smoking, drinking, drugs and medication use, alcohol),^4^ sleep pattern,^5^ daily sport activities,^19^, ^20^ stress level,^3^ coffee consumption,^3^ food quality,^6^ and antioxidants use.^17^, ^18^, ^21^, ^22^

Since patient does not smoke or use drugs, only two habits that he changed in this period are reducing alcohol consumption form 4–5 drinks per week to 1–2 maximum. At the same time, he did not take any pain killers or anti‐inflammatory drugs during recovery period (or any other medications, just one listed in the Table 4).

With regard to the sleeping pattern, sports, and daily burned calories, significant changes have been implemented. Active training time and calories burned per day have been both increased by 50%, while sleeping pattern was increased by 20%. Unfortunately, there are not enough clinical studies and evidence that any of these parameters have positive influence on sperm quality.^5^, ^19^, ^20^

Stress and caffeine consumption was also investigated in the past;^3^ however as single or dual factors, there is no prove about direct influence on spermatogenesis. Patient is a manager in big global company at very stressful position, and he made decision that during this period of recovery he would take 5 weeks' vacation in the south of hemisphere (summertime in December and January), to be able to have more outdoor activities, fresh area, access to the beach and more relaxed daily time. Due to that he tried to have around 150 min per day of cardio exercises including walking and additional 70 min of weightlifting in the gym. Before recovery, he had 4–5 coffees per day and normally at least 1–2 beverages with caffeine. During recovery, he reduced coffee to 2 per day and no caffeine beverages at all.

Fresh food rich of antioxidants are strongly recommended for better sperm quality in male infertility. This theory is well known over the decades, and it does have positive influence on sperm count and motility.^6^, ^17^, ^18^, ^21^, ^22^ Normally, patient would have 3 major meals per day (breakfast, lunch, and dinner), and one small snack (fruits, nuts, pastries) in the afternoon. During the recovery period, he added one more snack between breakfast and lunch, and both snacks have been focusing fresh fruits and/or vegetables as a meal. For the lunch and dinner, he also increased his meals for 20%–25% (due to higher calories needed), which was all covered by vegetables. In the period of vacation (in Brazil), he did have everyday portion of 300 mL acai, which is very well know super‐antioxidant.^24^ Clinical trials have suggested that acai can protect against metabolic stress induced by oxidation, inflammation, vascular abnormalities, and physical exertion. Due to its medicinal properties and the absence of undesirable effects, acai shows a promising future in health promotion and disease prevention, including also male infertility.^24^

Obesity in combination with aging and potentially diabetes could be a problem in male low sperm count. However, in this case, patient was having healthy situation with fat percentage between 11 and 18, and muscular percentage of the body between 42 and 49 (Figure 1). Body mass index was slightly higher (28.6–29.6) with his weight between 95 and 98 kg and height of 182 cm (Figure 1). However, overall, his status was healthy, sporty, masculine, and muscular mature man in early 40s.

And finally, the last big portion of the recovery process was related to use of food supplements, mostly antioxidants, which are listed in the Table 4. In the literature, there are many clinical studies with Vitamin E, Vitamin C, coenzyme Q10, selenium, zinc, folic acid, Vitamin D, multivitamins and multimineral products, herbal medications, omega‐3, and amino acids;^6^, ^17^, ^18^, ^21^, ^22^ however, the best approach and recommendation were to use it combined. Therefore, patient use that approach with clear starting awareness that high doses of some supplements would not be good for liver or kidney over longer period than 3–4 months. Accordingly, spermatogenesis and blood tests intermediate check point were done after 2.5 months to see whether there are any significant side effects on organs and improvement from sperm quality. In the Table 3 at T2 which was 2.5 months after treatment started sperm quality significantly increased and it is almost compared to the results from previous studies after medical treatment or at least 6 months.^15^ With these results, it was already possible to proceed with IVF approach. However, patient was keeping habits and therapy for 1 month more (T3) and reached completely healthy level of 15.5 million sperms per milliliter and total 46.5 million sperms in one sampling. All other spermatogenesis parameters also significantly improved (Table 3). At the same time point (T3), all blood tested parameters also got into the normal range and patient was producing his own testosterone (527 ng/dL).

## 4 CONCLUSION

This study case used data from 44‐year‐old (November 2022) healthy man who managed to improve sperm count from zero to healthy level in less than 4 months after several steroids' cycles. These findings raise the possibility of fast recovery for men who are in reproductive age and at the same time use steroids as bodybuilding supplements. Therapy, change of habits, and sport activities for limited time can speed up the recovery process for spermatogenesis in healthy men with very successful outcome. However, it must be clear that this was only one case with extreme and unique extraordinary outcome. Therefore, it is exception and not the rule, until proven different. Additional trials on bigger subject group would be needed to confirm multiple parameters approach usability, as suggested in this paper. It would be also very important to select right subjects for future evaluation which should be healthy men and with history of bodybuilding supplements use (e.g., steroids).

## AUTHOR CONTRIBUTIONS

**Rade Injac:** Conceptualization; data curation; formal analysis; funding acquisition; investigation; methodology; project administration; resources; software; supervision; validation; visualization; writing – original draft; writing – review and editing.

## FUNDING INFORMATION

None.

## CONFLICT OF INTEREST STATEMENT

The author declares no conflicts of interest.

## ETHICAL APPROVAL

Approved (No. 2022/M/32405).

## CONSENT

Written information consent was also obtained from the patient to publish this report in accordance with the “journal's patient consent policy.”

## Data Availability

Request for data would be considered upon request to the corresponding author.
